# Universality of maximum-work efficiency of a cyclic heat engine based on a finite system of ultracold atoms

**DOI:** 10.1038/s41598-017-06615-z

**Published:** 2017-07-24

**Authors:** Zhuolin Ye, Yingying Hu, Jizhou He, Jianhui Wang

**Affiliations:** 1 Department of Physics, Nanchang University, Nanchang, 330031 China; 2 Department of Chemistry and Biochemistry, University of Maryland, College Park, MD 20742 USA; 3 State Key Laboratory of Theoretical Physics, Institute of Theoretical Physics, Chinese Academy of Sciences, Beijing, 100190 China

## Abstract

We study the performance of a cyclic heat engine which uses a small system with a finite number of ultracold atoms as its working substance and works between two heat reservoirs at constant temperatures *T*_*h*_ and *T*_*c*_(<*T*_*h*_). Starting from the expression of heat capacity which includes finite-size effects, the work output is optimized with respect to the temperature of the working substance at a special instant along the cycle. The maximum-work efficiency *η* ^*m*w^ at small relative temperature difference can be expanded in terms of the Carnot value ${{\boldsymbol{\eta }}}_{{\boldsymbol{C}}}={\bf{1}}-{{\boldsymbol{T}}}_{{\boldsymbol{c}}}/{{\boldsymbol{T}}}_{{\boldsymbol{h}}}$, ${{\boldsymbol{\eta }}}^{{\boldsymbol{m}}{\bf{w}}}={{\boldsymbol{\eta }}}_{{\boldsymbol{C}}}/{\bf{2}}+{{\boldsymbol{\eta }}}_{{\boldsymbol{C}}}^{{\bf{2}}}({\bf{1}}/{\bf{8}}+{{\boldsymbol{a}}}_{{\bf{0}}})+{\boldsymbol{\ldots }}$, where *a*_0_ is a function depending on the particle number *N* and becomes vanishing in the symmetric case. Moreover, we prove using the relationship between the temperatures of the working substance and heat reservoirs that the maximum-work efficiency, when accurate to the first order of *η*_*C*_, reads ${{\boldsymbol{\eta }}}^{{\boldsymbol{m}}{\bf{w}}}={{\boldsymbol{\eta }}}_{{\boldsymbol{CA}}}+{\bf{O}}$(Δ*T* ^2^). Within the framework of linear irreversible thermodynamics, the maximum-power efficiency is obtained as ${{\boldsymbol{\eta }}}^{{\boldsymbol{mp}}}={{\boldsymbol{\eta }}}_{{\boldsymbol{CA}}}+{\bf{O}}$(Δ*T* ^2^) through appropriate identification of thermodynamic fluxes and forces, thereby showing that this kind of cyclic heat engines satisfy the tight-coupling condition.

## Introduction

Heat engines (pumps) and refrigerators, converting thermal energy into mechanical work and vice versa, play an excellent platform for studying the thermodynamics of the systems driven out of equilibrium due to the interaction with work sources or heat baths, in addition to their potential energy applications in society. The working fluid for a macroscopic thermal engine is typically a system which contains on the order of 10^24^ particles. Advanced experimental techniques in recent years have led to miniaturization of heat devices where the working fluid is a mesoscopic or microscale system with quite a few number of particles (or with even one single particle)^1–3^. Among them, one prominent example is a heat engine based on an ultracold atom system^2^. Meanwhile, theoretical descriptions of thermodynamics of a thermal engine based on an ideal or interacting small system far away from the thermodynamic limit have been intensively studied^4–13^.

One of the most important issues on the topic of thermodynamics of heat engines is the study of their performance characteristics within the context of finite-time thermodynamics^14–20^, which began with a seminal paper by Curzon and Ahlborn^14^. Based on the endoreversible assumption, Curzon and Ahlborn found using the Newton’s heat transfer law that the maximum-power efficiency *η* ^*mp*^ of a finite-time Carnot-like cycle, working between a hot and a cold reservoir at constant temperatures *T*_*h*_ and *T*_*c*_(<*T*_*h*_), is given by the following Curzon-Ahlborn (CA) efficiency: ${\eta }_{CA}=1-\sqrt{{T}_{c}/{T}_{h}}$, with universality at small differences of relative temperature (linear response regime),

1 $${\eta }_{CA}=\frac{{\eta }_{C}}{2}+\frac{{\eta }_{C}^{2}}{8}+{\mathscr{O}}({\eta }_{C}^{3}),$$

where ${\eta }_{C}=1-{T}_{c}/{T}_{h}$ is the Carnot efficiency. Intensive studies have been subsequently presented on the finite-power performance of various types of classical or quantum heat engines, with particular emphasis on the possibly universal bounds of the maximum-power efficiency^4, 11, 21–42^. Among them, the issue of how microscale and macroscale heat devices behave in their performance^23, 25, 29, 30, 32, 34, 38–40^ has been extensively addressed, which demonstrates that for some heat engine models there exist certain sort of universality of the CA efficiency. On the other hand, the issue of maximum-work efficiency in reversible heat engines such as the Otto, Brayton, Diesel, and Atkinson cycle, has also attracted much attention^43–46^, with special emphasis of comparison with the CA efficiency *η*_*CA*_ [cf. Eq. (1)]. While working in the maximum-work regime, a reversible cyclic heat engine can be mapped into a cycle in which the working substance interacts with an infinite number of auxiliary reservoirs, and it necessarily has efficiency below the Carnot efficiency *η*_*C*_. The CA efficiency was also observed for a certain class of reversible heat engines performing at maximum-work regime^43–46^.

Despite much progress in this research field, so far, no unified thermodynamic description of the performance of general two-heat-source engines working in the maximum-work regime, particularly microscale or mesoscale heat engines, is available. Here we raise several questions: (1) are there any finite-size effects on the maximum-work efficiency? what the effects if there are? (2) Is there a certain sort of universality for the maximum-work efficiency in a reversible cycle (like maximum-power efficiency in an irreversible cycle)? if yes, to what extent the bounds of the maximum-work efficiency are universal? (3) what is the connection between the maximum-work efficiency for a reversible heat engine and the maximum-power efficiency for an irreversible one? To answer these questions, we analyze the maximum-work efficiency of a cyclic heat engine whose working substance consists of an arbitrary number of ultracold atoms confined in a trapping potential. We find that the maximum-work efficiency can be expanded as a *N* – dependent function: ${\eta }^{mw}={\eta }_{C}/2+(1/8+{a}_{0})\,{\eta }_{C}^{2}+\cdots$, with *a*_0_ being *N* – dependent parameter, which reduces to the size-independent universality ${\eta }_{C}/2+{\eta }_{C}^{2}/8$ in the symmetric case. By establishing the linkage between the temperatures of the working substance and heat reservoirs, we prove that the maximum-work efficiency is given by ${\eta }^{m{\rm{w}}}={\eta }_{C}/2+{\mathscr{O}}({\eta }_{C}^{2})$ in the linear response regime. Employing the linear irreversible thermodynamics, we show that the maximum-power efficiency is of the form ${\eta }^{mp}={\eta }_{CA}+{\mathscr{O}}\,({\rm{\Delta }}{T}^{2})$ as the cyclic heat engines satisfy the strong-coupling condition.

## Results

### Cyclic heat engine based on a small system

#### Heat capacity for a small system

The density of states *ρ*(*ε*) for a system with its energy *ε* can be determined according to the number of states *ν*(*ε*), for which the energy is bounded from above by a given energy *ε*. The number of states *ν*(*ε*) in a *d* – dimensional system is exactly equal to the sum of the number of points of a *d* – dimensional lattice with lattice constants $\hslash {\omega }_{i}(i=1,2,\ldots ,d)$ involved both inside the volume and on the surface area of the simplex described by $\{{x}_{i}\ge 0,{\sum }_{i=1}^{d}\,{x}_{i}\le \varepsilon \}$, with ${x}_{i}=\hslash {\omega }_{i}$ ^47–51^. For a finite system of Bosons, which was also used to experimentally realize Bose-Einstein condensation^52–54^, the number of the sates on the surface of the simplex can not be neglected, thereby indicating that the term depicting the states on the surface could be included in the expression of *ν*(*ε*). Accordingly, one can parameterize the density of states^48–51^ by ($\hslash \equiv 1$)

2 $$\rho (\varepsilon )={\varphi }_{1}\frac{{\varepsilon }^{d-1}}{{\omega }^{d}}+{\varphi }_{2}\frac{{\varepsilon }^{d-2}}{{\omega }^{d-1}},$$

where we have defined $\omega \equiv {\prod }_{i=1}^{d}\,{({\omega }_{i})}^{\mathrm{1/}d}$, and the coefficients *ϕ*_1_ and *ϕ*_2_ depend on the nature of the trapping potential. The second term in Eq. (2) arises from the contribution of the surface states and *ϕ*_2_ tends to be vanishing for a macroscopic system approaching the thermodynamic limit.

For a Bose system, the average particle occupation *n*(*ε*) for a system in contact with a heat reservoir at temperature *T* is given by (${k}_{B}\equiv 1$), $n(\varepsilon )={[{e}^{(\varepsilon -\mu )/T}-\mathrm{1]}}^{-1}$, where *μ* is the chemical potential. The total number of ultracold atoms *N* and the total system energy *E* can be expressed as, $N=\int \rho (\varepsilon )n(\varepsilon )d\varepsilon$ and $E=\int \rho (\varepsilon )n(\varepsilon )\varepsilon d\varepsilon$, respectively. Without going through a detailed derivation, one can find combining these two expressions of *N* and *E* that, the heat capacity $[{C}^{\{\iota \}}(N)={\tfrac{\partial E(N)}{\partial T}|}_{\iota ={\rm{const}}}]$ for a Bose system at low temperatures below transition temperature undergoing an isochoric ($\iota ={\rm{volume}}$) or an isobaric (..) process, can be expressed in the form of refs 49 and 50

3 $${C}^{\{\iota \}}(N)=\xi {T}^{\alpha }+{\xi }^{0}{N}^{\gamma }{T}^{\alpha -1},$$

where *ξ* and *ξ* ^0^ are constants independent of system size (or particle number *N*). Here both the parameter *α* and the negative parameter *γ*(<0) depend on the nature of the trapping potential and the process the system undergoes. It is clear that the second term in Eq. (3) is the correction accounting for the effects induced by finite size of the system and it must be vanishing when the particle number *N* approaches infinity. As a very simple example, we consider an ideal Bose gas confined in a three-dimesional isotropic harmonic trap whose frequey $\omega ={\omega }_{1}={\omega }_{2}={\omega }_{3}$ scales as $\omega \sim {V}^{-\mathrm{1/3}}$ with *V* being the volume of the trap. Then the density of states^49, 50^ reads $\rho (\varepsilon )={\varepsilon }^{2}/2+\varphi \varepsilon$, where the paramter *ϕ* depends on the form of the potential. Then the direct calcuation of $N={\int }_{0}^{\infty }\,\rho (\varepsilon )n(\varepsilon )d\varepsilon$ and $E={\int }_{0}^{\infty }\,\rho (\varepsilon )n(\varepsilon )\varepsilon d\varepsilon$, where $n(\varepsilon )=1/[{e}^{(\varepsilon -\mu )/T}-1]$, yields the particle number $N={N}_{0}+{T}^{3}{g}_{3}(z)+\varphi {T}^{2}{g}_{2}(z)$ and the total energy $E={E}_{0}+3{T}^{4}{g}_{4}(z)+2\varphi {T}^{3}{g}_{3}(z)$. Here the Bose-Einstein function *g*_*j*_(*z*) is determined by ${g}_{j}(z)=\frac{1}{{\rm{\Gamma }}(j)}{\int }_{0}^{\infty }\,\frac{{x}^{j-1}}{{z}^{-1}{e}^{x}-1}$, where $z=\exp \,(\mu /T)$ denotes the fugacity. At the crtical point *N*_0_ = 0 and *μ* = 0 (*z* = 1), one can obtain the transition temperature ${T}_{c}(N)={T}_{0}\,[1-\frac{\zeta \mathrm{(2)}}{3\zeta {\mathrm{(3)}}^{\mathrm{2/3}}}\varphi {N}^{-\mathrm{1/3}}]$, where the use of $\zeta (j)\equiv {g}_{j}\mathrm{(1)}$ has been made. Here the transition temperature in the thermodynamic limit, ${T}_{0}=\hslash \omega {[N/\zeta \mathrm{(3)}]}^{\mathrm{1/3}}$, as a reference value can be assumed to be *T*_0_ = 1, since the density of paritcles is kept constant (as realized in the experiment^52, 53^). Combining these two expressions of *N* and *E* gives rise to the values *T* and *μ*. Throughout the paper we use the fugacity *z* = 1 by assuming the temperature to be lower than its transition value. Using the definition ${C}^{\{\iota \}}(N)=\partial E/\partial T$, we then arrive at a special form of Eq. (3) in which $\xi =12\zeta \mathrm{(4)}/\zeta \mathrm{(3)}$, ${\xi }^{0}=6\varphi \zeta {\mathrm{(3)}}^{\mathrm{1/3}}$, *α* = 3, and *γ* = −1/3.

#### Work and efficiency

**Figure 1:**
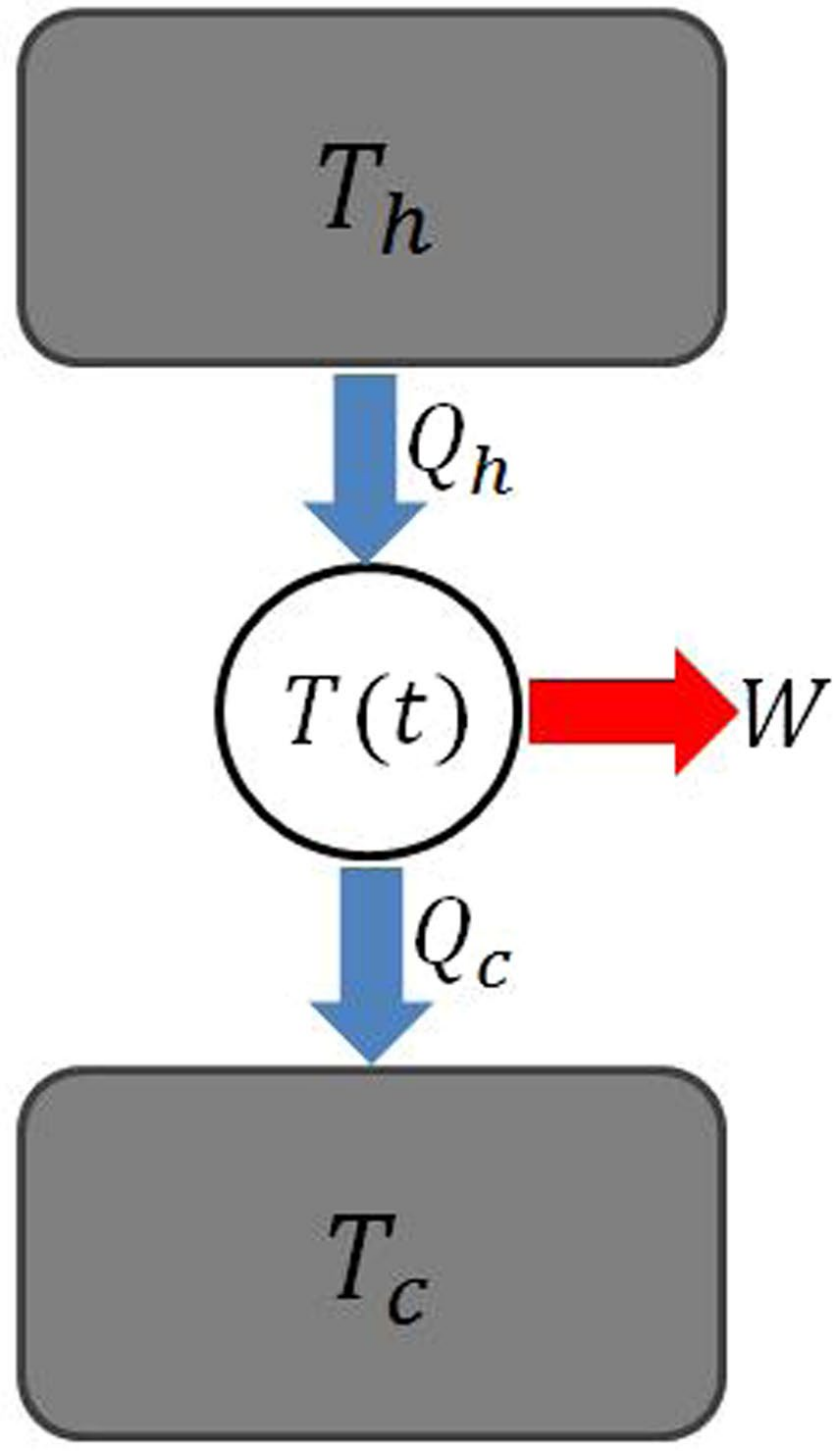
Graphic sketch of a two-heat-source machine.

We now consider a general two-heat-source heat engine, which may be Carnot cycle, Brayton cycle, Diesel Cycle, or Otto cycle, etc^55^. The diagram of such a heat engine is illustrated in Fig. 1. The cyclic heat engine gets the working subsystem back to its original state at the end of each cycle.

By integrating heat capacity ${C}^{\{\iota \}}$ (we will use *C* instead of ${C}^{\{\iota \}}$ for simplicity) in Eq. (3) over the temperature *T*, we find that the heats absorbed and released by the working substance in the hot and cold thermodynamic processes are respectively given by

4 $$\begin{array}{l}{Q}_{h}={\int }_{{T}_{hw}}^{{T}_{h}}\,{C}_{h}dT={\xi }_{h}{\int }_{{T}_{hw}}^{{T}_{h}}\,{T}^{\alpha }dT+{\xi }_{h}^{0}{N}^{\gamma }{\int }_{{T}_{hw}}^{{T}_{h}}\,{T}^{\alpha -1}dT,\end{array}$$

5 $$\begin{array}{l}{Q}_{c}={\int }_{{T}_{cw}}^{{T}_{c}}\,{C}_{c}dT={\xi }_{c}{\int }_{{T}_{cw}}^{{T}_{c}}\,{T}^{\alpha }dT+{\xi }_{c}^{0}{N}^{\gamma }{\int }_{{T}_{cw}}^{{T}_{c}}\,{T}^{\alpha -1}dT,\end{array}$$

where *T*_*h*,*c*_ is the temperature of the hot or cold reservoir, and ${T}_{hw,cw}$ is the minimum (maximum) value of the temperature of the working substance along the heat-transfer process. Direct calculation of Eqs (4) and (5) yields

6 $${Q}_{h}=\frac{{\xi }_{h}}{\alpha +1}\,({{T}_{h}}^{\alpha +1}-{T}_{hw}^{\alpha +1})+\frac{{\xi }_{h}^{0}{N}^{\gamma }}{\alpha }\,({{T}_{h}}^{\alpha }-{T}_{hw}^{\alpha }),$$

7 $${Q}_{c}=\frac{{\xi }_{c}}{\alpha +1}\,({T}_{c}^{\alpha +1}-{T}_{cw}^{\alpha +1})+\frac{{\xi }_{c}^{0}{N}^{\gamma }}{\alpha }\,({T}_{c}^{\alpha }-{T}_{cw}^{\alpha }).$$

As there is no net change in the internal energy after every single cycle, the work produced by the heat engine in a cycle, $W={Q}_{h}+{Q}_{c}$, and the efficiency $\eta =1+{Q}_{c}/{Q}_{h}$ are, respectively, given by

8 $$W=\frac{{\xi }_{h}}{\alpha +1}\,({{T}_{h}}^{\alpha +1}-{T}_{hw}^{\alpha +1})+\frac{{\xi }_{h}^{0}{N}^{\gamma }}{\alpha }\,({{T}_{h}}^{\alpha }-{T}_{hw}^{\alpha })+\frac{{\xi }_{c}}{\alpha +1}\,({T}_{c}^{\alpha +1}-{T}_{cw}^{\alpha +1})+\frac{{\xi }_{c}^{0}{N}^{\gamma }}{\alpha }\,({T}_{c}^{\alpha }-{T}_{cw}^{\alpha }),$$

9 $$\eta =1+\frac{{\xi }_{c}\alpha ({T}_{c}^{\alpha +1}-{T}_{cw}^{\alpha +1})+{\xi }_{c}^{0}{N}^{\gamma }(\alpha +\mathrm{1)}({T}_{c}^{\alpha }-{T}_{cw}^{\alpha })}{{\xi }_{h}\alpha ({{T}_{h}}^{\alpha +1}-{T}_{hw}^{\alpha +1})+{\xi }_{h}^{0}{N}^{\gamma }(\alpha +\mathrm{1)}({{T}_{h}}^{\alpha }-{T}_{hw}^{\alpha })}.$$

### Maximum-work efficiency

#### Optimization on the work under the reversible assumption

The average entropy change per cycle stems solely from heat exchange between the system and the baths. The total change in the entropy after a single cycle must be vanishing, i.e.,

10 $${\rm{\Delta }}{S}_{cycle}={\rm{\Delta }}{S}_{h}+{\rm{\Delta }}{S}_{c}=0,$$

where Δ*S*_*h*,*c*_ is the change in entropy along the hot or cold process. Assuming that the cycle is reversible, Eq. (10) can be re-expressed as

11 $${\rm{\Delta }}{S}_{cycle}={\int }_{{T}_{hw}}^{{T}_{h}}\,\frac{{C}_{h}}{T}dT+{\int }_{{T}_{cw}}^{{T}_{c}}\,\frac{{C}_{c}}{T}dT=\mathrm{0,}$$

leading to the equation,

12 $$\begin{array}{rcl}0={\rm{\Delta }}{S}_{cycle} & = & \frac{{\xi }_{h}}{\alpha }\,({{T}_{h}}^{\alpha }-{T}_{hw}^{\alpha })+\frac{{\xi }_{h}^{0}{N}^{\gamma }}{\alpha -1}\,({{T}_{h}}^{\alpha -1}-{T}_{hw}^{\alpha -1})\\  &  & +\frac{{\xi }_{c}}{\alpha }\,({T}_{c}^{\alpha }-{T}_{cw}^{\alpha })+\frac{{\xi }_{c}^{0}{N}^{\gamma }}{\alpha -1}\,({T}_{c}^{\alpha -1}-{T}_{cw}^{\alpha -1}).\end{array}$$

We now turn to the optimization on the heat engine by maximizing the work output. As the total change in the entropy of the system must be vanishing after a full cycle, maximizing the work output equivalent to maximizing the Lagrangian function:

13 $$L=W-\lambda {\rm{\Delta }}{S}_{cycle},$$

where *λ* is the Lagrange multiplier. Substituting Eqs (8) and (12) into Eq. (13), and setting $\frac{\partial L}{\partial {T}_{hw}}=0$ and $\frac{\partial L}{\partial {T}_{cw}}=0$, we can readily obtain

14 $${T}_{hw}^{\ast }={T}_{cw}^{\ast }.$$

On substitution of Eq. (14) into Eq. (12), we obtain the equation

15 $${T}_{hw}^{\ast \alpha }+\frac{\alpha {N}^{\gamma }({\xi }_{c}^{0}+{\xi }_{h}^{0})}{(\alpha -1)({\xi }_{c}+{\xi }_{h})}{T}_{hw}^{\ast \alpha -1}-[\frac{\alpha {N}^{\gamma }({\xi }_{c}^{0}{T}_{c}^{\alpha -1}+{\xi }_{h}^{0}{T}_{h}^{\alpha -1})}{(\alpha -1)({\xi }_{c}+{\xi }_{h})}+\frac{{\xi }_{c}{T}_{c}^{\alpha }+{\xi }_{h}{T}_{h}^{\alpha }}{{\xi }_{c}+{\xi }_{h}}]=0.$$

Equation (15) shows that the optimal values of ${T}_{hw}^{\ast }$ $({T}_{cw}^{\ast })$ at maximum work depends on the ratio parameters ${r}_{1}={\xi }_{c}/{\xi }_{h}$, ${r}_{2}={\xi }_{c}^{0}/{\xi }_{h}^{0}$, and ${r}_{3}={\xi }_{h}^{0}/({\xi }_{h}{T}_{c})$, dimensionality *d* (described by *α*), and particle number *N*. In principle, we can determine numerically the efficiency at maximum work, ${\eta }_{e{\rm{x}}}^{m{\rm{w}}}$, by inserting Eq. (15) into Eq. (9). However, mathematically, it is not likely to find an exact analytical expression for the maximum-work efficiency in Eq. (9) for *α* ≥ 5 according to Abel-Ruffini theorem. That means, in order to consider the general case, we have to resort to an approximation method for finding the analytical solution to Eq. (15).

In order to derive an analytic result, we substitute ${T}_{hw}^{\ast }={T}_{h}-{\rm{\Delta }}{T}_{w}^{h\ast }$ into Eq. (15) and expand that with respect to ${\rm{\Delta }}{T}_{w}^{h\ast }$ to obtain

16 $$\begin{array}{l}\frac{{\xi }_{c}({T}_{h}^{\alpha }-{T}_{c}^{\alpha })}{{\xi }_{c}+{\xi }_{h}}+\frac{{\xi }_{c}^{0}\alpha {N}^{\gamma }({T}_{h}^{\alpha -1}-{T}_{c}^{\alpha -1})}{({\xi }_{c}+{\xi }_{h})(\alpha -1)}\\ \quad -\alpha {T}_{h}^{\alpha -1}[1+\frac{\alpha {N}^{\gamma }({\xi }_{c}^{0}+{\xi }_{h}^{0})}{{T}_{h}({\xi }_{c}+{\xi }_{h})}]\,{\rm{\Delta }}{T}_{w}^{h\ast }+{\mathscr{O}}({\rm{\Delta }}{T}_{w}^{h\ast 2})=\mathrm{0,}\end{array}$$

which yields the optimal values of ${T}_{hw}^{\ast }$ $({T}_{cw}^{\ast })$

17 $${T}_{hw}^{\ast }={T}_{cw}^{\ast }={T}_{h}\{1+\frac{{\xi }_{c}{T}_{h}(\alpha -1)({T}_{c}^{\alpha }{T}_{h}^{-\alpha }-1)+{\xi }_{c}^{0}\alpha {N}^{\gamma }({T}_{c}^{\alpha -1}{T}_{h}^{1-\alpha }-1)}{\alpha (\alpha -1)[{N}^{\gamma }({\xi }_{c}^{0}+{\xi }_{h}^{0})+{T}_{h}({\xi }_{c}+{\xi }_{h})]}\}.$$

When we combine the identity $-\frac{{\xi }_{h}\,(\alpha -1)\,({{T}_{h}}^{\alpha }-{T}_{hw}^{\alpha })+{\xi }_{h}^{0}{N}^{\gamma }\alpha \,({{T}_{h}}^{\alpha -1}-{T}_{hw}^{\alpha -1})}{{\xi }_{c}\,(\alpha -1)\,({T}_{c}^{\alpha }-{T}_{cw}^{\alpha })+{\xi }_{c}^{0}{N}^{\gamma }\alpha \,({T}_{c}^{\alpha -1}-{T}_{cw}^{\alpha -1})}=1$, which was derived from Eq. (12), and the expression of efficiency *η* given by Eq. (9), we find that the efficiency can be rewritten as

18 $$\begin{array}{rcl}\eta  & = & 1-\frac{{\xi }_{c}\alpha ({T}_{c}^{\alpha +1}-{T}_{cw}^{\alpha +1})+{\xi }_{c}^{0}{N}^{\gamma }(\alpha +\mathrm{1)}({T}_{c}^{\alpha }-{T}_{cw}^{\alpha })}{{\xi }_{h}\alpha ({{T}_{h}}^{\alpha +1}-{T}_{hw}^{\alpha +1})+{\xi }_{h}^{0}{N}^{\gamma }(\alpha +\mathrm{1)}({{T}_{h}}^{\alpha }-{T}_{hw}^{\alpha })}\\  &  & \times \,\frac{{\xi }_{h}(\alpha -1)({{T}_{h}}^{\alpha }-{T}_{hw}^{\alpha })+{\xi }_{h}^{0}{N}^{\gamma }\alpha ({{T}_{h}}^{\alpha -1}-{T}_{hw}^{\alpha -1})}{{\xi }_{c}(\alpha -1)({T}_{c}^{\alpha }-{T}_{cw}^{\alpha })+{\xi }_{c}^{0}{N}^{\gamma }\alpha ({T}_{c}^{\alpha -1}-{T}_{cw}^{\alpha -1})}.\end{array}$$

A result of substituting Eq. (17) into Eq. (18) is that the maximum-work efficiency output turns out to be

19 $$\begin{array}{rcl}{\eta }^{m{\rm{w}}} & = & 1-\frac{{\xi }_{c}\alpha ({T}_{c}^{\alpha +1}-{{\rm{\Lambda }}}^{\alpha +1})+{\xi }_{c}^{0}{N}^{\gamma }(\alpha +\mathrm{1)}({T}_{c}^{\alpha }-{{\rm{\Lambda }}}^{\alpha })}{{\xi }_{h}\alpha ({{T}_{h}}^{\alpha +1}-{{\rm{\Lambda }}}^{\alpha +1})+{\xi }_{h}^{0}{N}^{\gamma }(\alpha +\mathrm{1)}({{T}_{h}}^{\alpha }-{{\rm{\Lambda }}}^{\alpha })}\\  &  & \times \,\frac{{\xi }_{h}(\alpha -1)({{T}_{h}}^{\alpha }-{{\rm{\Lambda }}}^{\alpha })+{\xi }_{h}^{0}{N}^{\gamma }\alpha ({{T}_{h}}^{\alpha -1}-{{\rm{\Lambda }}}^{\alpha -1})}{{\xi }_{c}(\alpha -1)({T}_{c}^{\alpha }-{{\rm{\Lambda }}}^{\alpha })+{\xi }_{c}^{0}{N}^{\gamma }\alpha ({T}_{c}^{\alpha -1}-{{\rm{\Lambda }}}^{\alpha -1})},\end{array}$$

where ${\rm{\Lambda }}\equiv {T}_{h}\,\{1+\frac{{\xi }_{c}{T}_{h}\,(\alpha -1)\,({T}_{c}^{\alpha }{T}_{h}^{-\alpha }-1)+{\xi }_{c}^{0}\alpha {N}^{\gamma }\,({T}_{c}^{\alpha -1}{T}_{h}^{1-\alpha }-1)}{\alpha \,(\alpha -1)\,[{N}^{\gamma }\,({\xi }_{c}^{0}+{\xi }_{h}^{0})+{T}_{h}\,({\xi }_{c}+{\xi }_{h})]}\}$ has been adopted.

**Figure 2:**
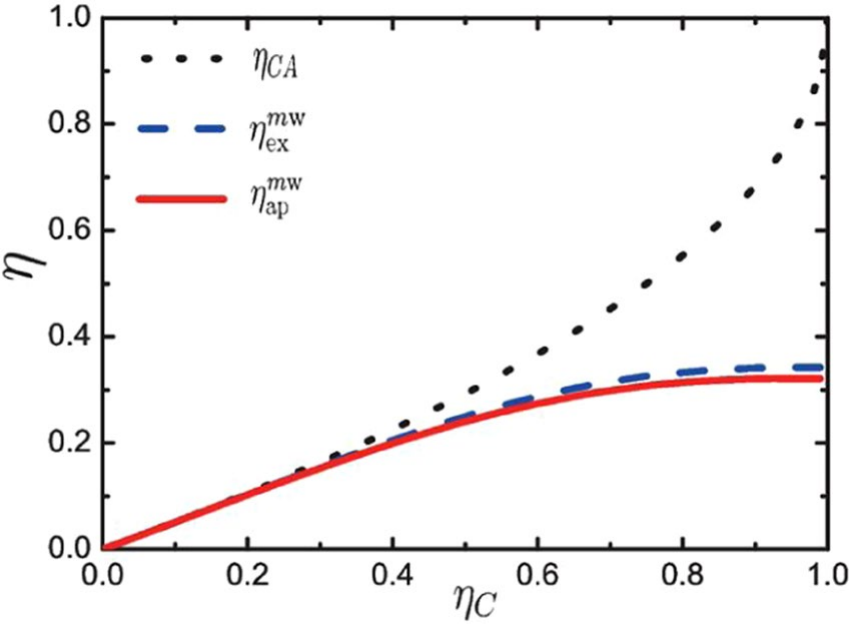
The maximum-work efficiency *η* ^*m*w^ or the CA efficiency *η*_*CA*_ as a function of the Carnot efficiency *η*_*C*_. The approximate and exact results of the optimal efficiency, ${\eta }_{{\rm{ap}}}^{m{\rm{w}}}$ and ${\eta }_{{\rm{ex}}}^{m{\rm{w}}}$ are denoted by a blue dashed line and a red solid line, respectively, while the CA efficiency *η*_*CA*_ is represented by a black dotted line. Here the parameters are *N* = 100, *α* = 3, *γ* = −3, and ${r}_{1}={r}_{2}={r}_{3}=1$.

To compare with the approximate formula Eq. (19), for instance, we calculate the exact values .. for the heat engine working with a Bose system with *N* = 100 particles confined in a three-dimensional isotropic harmonic trap where *α* = 3 and *γ* = −3. In Fig. 2 we compare the approximate result ${\eta }_{{\rm{ap}}}^{m{\rm{w}}}$ obtained from Eq. (19), red solid line, with the exact values values ${\eta }_{{\rm{ex}}}^{m{\rm{w}}}$, blue dashed line, and the CA efficiency, black dotted line. We see from Fig. 2 that the approximate result is in nice agreement with the exact one, providing a strong argument in favor of our approach. Another point we note from Fig. 2 is that our result coincides with the CA efficiency at very small relative temperature difference (or at small values of *η*_*C*_).

Equation (19) can be expanded to be valid up to quadratic order in *η*_*C*_ by using ${r}_{1}={\xi }_{c}/{\xi }_{h}$, ${r}_{2}={\xi }_{c}^{0}/{\xi }_{h}^{0}$, and ${r}_{3}={\xi }_{h}^{0}/({\xi }_{h}{T}_{c})$ as

20 $${\eta }^{m{\rm{w}}}=\frac{{\eta }_{C}}{2}+(\frac{1}{8}+{a}_{0})\,{\eta }_{C}^{2}+{\mathscr{O}}({\eta }_{C}^{3}),$$

where

$$\begin{array}{c}{a}_{0}=\{{r}_{1}^{2}\,({r}_{1}+3{r}_{2}{r}_{3}{N}^{\gamma })\,[2\alpha +1+{r}_{3}{N}^{\gamma }\,(2\alpha -1)]\\ \qquad \,+\,{r}_{2}{r}_{3}{N}^{\gamma }\{[{r}_{3}^{3}{N}^{3\gamma }({r}_{2}^{2}-1)-3{r}_{3}^{2}{N}^{2\gamma }-3{r}_{3}{N}^{\gamma }-1]+{r}_{2}^{2}{r}_{3}^{2}{N}^{2\gamma }\,(2\alpha +1)\}\\ \qquad \,-\,{r}_{1}\{{r}_{3}{N}^{\gamma }\,(2\alpha +1)\,[{r}_{3}{N}^{\gamma }(3-3{r}_{2}^{2}+{r}_{3}{N}^{\gamma })+3]\\ \qquad \,+(2\alpha +1)-3{r}_{2}^{2}{r}_{3}^{3}{N}^{3\gamma }\,(2\alpha -1)\}\}\\ \,/\{24\,\mathrm{(1}+{r}_{3}{N}^{\gamma })\,({r}_{1}+{r}_{2}{r}_{3}{N}^{\gamma })\,{\mathrm{(1}+{r}_{1}+{r}_{3}{N}^{\gamma }+{r}_{2}{r}_{3}{N}^{\gamma })}^{2}\}.\end{array}$$

Here the parameter *a*_0_ is dependent on the particle number (which corresponds to the system size). While the universality of the coefficient 1/2 in the linear term is recovered, the value of the coefficient for the quadratic term is dependent on the model parameters, such as the dimensionality, the system size (particle number), and the form of trapping potential, etc. To estimate the finite-size effects on the efficiency at maximum work, we consider two limits of system size, i.e., the particle number *N* = 1 and $N\to \infty$, which are as follows:

1. In the thermodynamic limit where $N\to \infty$ and thus *N* ^*γ*^ → 0, the parameter *a*_0_ simplifies to ${a}_{0}=({r}_{1}-1)$ $(2\alpha +\mathrm{1)}/[\mathrm{24(}{r}_{1}+1)]$, whose upper and lower bounds satisfy ${a}_{0}\le |\mathrm{(2}\alpha +\mathrm{1)}|/24$. As expected, the expression of *a*_0_ is completely independent of *r*_2_ and *r*_3_, since the term in Eq. (3), the correction to finite size of system, must be vanishing for the macroscopic system.
2. The minimization limit where *N* = 1 is considered to determine which range *a*_0_ should be situated in. For *r*_1_ → 0, while *r*_2_ → 0 leads to ${a}_{0}=\mathrm{(1}-2\alpha )/24$, ${r}_{2}\to \infty$ results in ${a}_{0}=\mathrm{(2}{r}_{3}\alpha -{r}_{3}+2\alpha -\mathrm{1)}/\mathrm{(24}{r}_{3}+\mathrm{24)}$, which simplifies to ${a}_{0}=\mathrm{(2}\alpha +\mathrm{1)}/24$ [${a}_{0}=\mathrm{(2}\alpha -\mathrm{1)}/24$] as *r*_3_ → 0 (${r}_{3}\to \infty$). It is therefore shown that, when *r*_1_ → 0, the value of *a*_0_ should satisfy $\mathrm{(1}-2\alpha )/24\le {a}_{0}\le \mathrm{(2}\alpha +\mathrm{1)}/24$ for *α* > 0 or $\mathrm{(2}\alpha -\mathrm{1)}/24\le$ ${a}_{0}\le \mathrm{(1}-2\alpha )/24$ for *α* < 0. For ${r}_{1}\to \infty$, *a*_0_ is independent of *r*_2_ and becomes ${a}_{0}=\mathrm{(2}\alpha +2{r}_{3}\alpha -{r}_{3}+$ $2\alpha +\mathrm{1)}/\mathrm{(24}{r}_{3}+\mathrm{24)}$, which simplifies to ${a}_{0}=\mathrm{(2}\alpha -\mathrm{1)}/24$ [${a}_{0}=\mathrm{(2}\alpha +\mathrm{1)}/24$] when ${r}_{3}\to \infty$ (*r*_3_ → 0). In a word, the minimization limit leads to $\mathrm{(1}-2\alpha )/24\le \alpha \le \mathrm{(2}\alpha +\mathrm{1)}/24$ for *α* > 0 and $\mathrm{(2}\alpha -\mathrm{1)}/24\le \alpha \le \mathrm{(1}-2\alpha )/24$ for *α* < 0.

Comparison between the two limits above shows the insensitive independence of the maximum-work efficiency on the particle number *N*, though the universality of the maximum-work efficiency is only valid up to the first order of *η*_*C*_. Quite interestingly, the symmetric scenario when *r*_1_ = *r*_2_ = 1 leads to vanishing *a*_0_ in Eq. (20), thereby showing that the universality of maximum-work efficiency is recovered,

21 $${\eta }^{m{\rm{w}}}=\frac{{\eta }_{C}}{2}+\frac{{\eta }_{C}^{2}}{8}+{\mathscr{O}}\,({\eta }_{C}^{3}),$$

which is independent of the parameters *r*_3_, *α* and *γ*, and particle number *N*. Hence, the universality, ${\eta }_{C}/2+{\eta }_{C}^{2}/8$, holds completely independently of the system size, the form and dimensionality of the trapping potential, and the system temperature, and of the interaction strength between particles^56^, as long as *r*_1_ = *r*_2_ = 1 which implies that the heat transfer coefficients in two heat-transfer processes are equal. We recover the coefficient of the second order term 1/8, which was derived from the heat engine model based on Newton’s heat transfer law^22^. It is, however, more general and indicating greater validity, because it was derived without a given heat-transfer law. The symmetric scenario we discussed here is similar to the symmetric fluxes for a steady heat engine^32^ and the symmetric dissipation for a cyclic heat engine^24^, as they share the same universal value of the quadratic coefficient for the efficiency at maximum power.

#### Maximum-work efficiency when accurate to the first order of *η*_*C*_

To study the nature of the universality of the maximum-work efficiency, we consider the linkage between the temperatures of the working substance and heat reservoirs in advance. We substitute ${T}_{h}=T+\frac{{\rm{\Delta }}T}{2}$, ${T}_{c}=T-\frac{{\rm{\Delta }}T}{2}$, ${T}_{hw}={T}_{w}+\frac{{\rm{\Delta }}{T}_{w}}{2}$ and ${T}_{cw}={T}_{w}-\frac{{\rm{\Delta }}{T}_{w}}{2}$, with ${\rm{\Delta }}{T}_{w}={T}_{hw}-{T}_{cw}$ and ${T}_{w}=\frac{{T}_{hw}+{T}_{cw}}{2}$, into Eq. (12), yielding

22 $$\begin{array}{l}\alpha {r}_{3}{N}^{\gamma }(T-\frac{{\rm{\Delta }}T}{2})\,[{(T+\frac{{\rm{\Delta }}T}{2})}^{\alpha -1}-{({T}_{w}+\frac{{\rm{\Delta }}{T}_{w}}{2})}^{\alpha -1}]\\ \quad +\,\alpha {r}_{2}{r}_{3}{N}^{\gamma }(T-\frac{{\rm{\Delta }}T}{2})\,[{(T-\frac{{\rm{\Delta }}T}{2})}^{\alpha -1}-{({T}_{w}-\frac{{\rm{\Delta }}{T}_{w}}{2})}^{\alpha -1}]\\ \quad +\,{r}_{1}(\alpha -1)\,[{(T-\frac{{\rm{\Delta }}T}{2})}^{\alpha }-{({T}_{w}-\frac{{\rm{\Delta }}{T}_{w}}{2})}^{\alpha }]\\ \quad +\,(\alpha -1)\,[{(T+\frac{{\rm{\Delta }}T}{2})}^{\alpha }-{({T}_{w}+\frac{{\rm{\Delta }}{T}_{w}}{2})}^{\alpha }]=0,\end{array}$$

where *r*_*i*_ with *i* = 1, 2, 3 were defined above Eq. (20). Here it can be observed from Eq. (22) that *T*_*w*_ is determined by *T*, Δ*T* and Δ*T*_*w*_ for given *r*_*i*_(*i* = 1, 2, 3), *N*, *γ*, and *α*. Without loss of generality, for the heat engine running in the linear response regime, we can therefore express *T*_*w*_ as the form:

23 $${T}_{w}={\chi }_{1}+{\chi }_{2}{\rm{\Delta }}T+{\chi }_{3}{\rm{\Delta }}{T}_{w}.$$

Inserting Eq. (23) into Eq. (22) and expanding it with respect to Δ*T* and Δ*T*_*w*_, we then arrive at

24 $$\begin{array}{l}\alpha {r}_{3}{N}^{\gamma }T(1+{r}_{2})\,({T}^{\alpha -1}-{\chi }_{1}^{\alpha -1})+(\alpha +1)\,(1+{r}_{1})\,({T}^{\alpha }-{\chi }_{1}^{\alpha })\,\{\frac{1}{2}\alpha (\alpha -1){T}^{\alpha -1}\\ \quad \times \,[{r}_{3}{N}^{\gamma }(1-{r}_{2})+1-{r}_{1}]-\frac{1}{2}\alpha {r}_{3}{N}^{\gamma }(1+{r}_{2})\,({T}^{\alpha -1}-{\chi }_{1}^{\alpha -1})-\alpha (\alpha -1)\,{\chi }_{2}{\chi }_{1}^{\alpha -2}\\ \quad \times \,[{r}_{3}{N}^{\gamma }T\mathrm{(1}+{r}_{2})+{\chi }_{1}(1+{r}_{1})]\}\,{\rm{\Delta }}T-\{\alpha (\alpha -1)\,{\chi }_{1}^{\alpha -2}[({\chi }_{1}+{r}_{3}{N}^{\gamma }T)\\ \quad \times \,({\chi }_{3}+\frac{1}{2})+({r}_{1}{\chi }_{1}+{r}_{2}{r}_{3}{N}^{\gamma }T)\,({\chi }_{3}-\frac{1}{2})]\}\,{\rm{\Delta }}{T}_{w}+{\mathscr{O}}({\rm{\Delta }}{T}^{2},{\rm{\Delta }}T{\rm{\Delta }}{T}_{w},{\rm{\Delta }}{T}_{w}^{2})=0.\end{array}$$

Because ${\rm{\Delta }}T={T}_{h}-{T}_{c}$ and ${\rm{\Delta }}{T}_{w}={T}_{hw}-{T}_{cw}$ are positive numbers, the corresponding coefficients must be equal to zero, which gives

25 $$\begin{array}{rcl}{\chi }_{1} & = & T,\\ {\chi }_{2} & = & \chi \equiv \frac{1}{2}\frac{{r}_{3}{N}^{\gamma }\mathrm{(1}-{r}_{2})+1-{r}_{1}}{{r}_{3}{N}^{\gamma }\mathrm{(1}+{r}_{2})+1+{r}_{1}},\\ {\chi }_{3} & = & -\chi =-\frac{1}{2}\frac{{r}_{3}{N}^{\gamma }\mathrm{(1}-{r}_{2})+1-{r}_{1}}{{r}_{3}{N}^{\gamma }\mathrm{(1}+{r}_{2})+1+{r}_{1}},\end{array}$$

yielding the simple form of Eq. (23),

26 $${T}_{w}=T+\chi {\rm{\Delta }}T-\chi {\rm{\Delta }}{T}_{w}.$$

This, with equations ${T}_{hw}={T}_{w}+\frac{{\rm{\Delta }}{T}_{w}}{2}$ and ${T}_{cw}={T}_{w}-\frac{{\rm{\Delta }}{T}_{w}}{2}$, gives rise to

27 $${T}_{hw}=T+\chi {\rm{\Delta }}T+(\frac{1}{2}-\chi )\,{\rm{\Delta }}{T}_{w},$$

28 $${T}_{cw}=T+\chi {\rm{\Delta }}T-(\frac{1}{2}+\chi )\,{\rm{\Delta }}{T}_{w}.$$

Substituting ${T}_{hw}={T}_{h}-{\rm{\Delta }}{T}_{w}^{h}$, ${T}_{h}=T+\frac{{\rm{\Delta }}T}{2}$ and ${T}_{c}=T-\frac{{\rm{\Delta }}T}{2}$, with ${\rm{\Delta }}T={T}_{h}-{T}_{c}$ and $T=\frac{{T}_{h}+{T}_{c}}{2}$, into Eq. (6), we find *Q*_*h*_ by expanding Eq. (6) with respect to ${\rm{\Delta }}{T}_{w}^{h}$ and Δ*T* to be

29 $${Q}_{h}=\frac{{\xi }_{c}{T}^{\alpha }\mathrm{(1}+{r}_{3}{N}^{\gamma })}{{r}_{1}}{\rm{\Delta }}{T}_{w}^{h}+{\mathscr{O}}\,({\rm{\Delta }}{T}_{w}^{h}{\rm{\Delta }}T,{\rm{\Delta }}{T}_{w}^{{h}^{2}}).$$

Similarly, directly inserting ${T}_{cw}={T}_{c}+{\rm{\Delta }}{T}_{w}^{c}$, ${T}_{h}=T+\frac{{\rm{\Delta }}T}{2}$ and ${T}_{c}=T-\frac{{\rm{\Delta }}T}{2}$ into Eq. (7), we expand Eq. (7) with respect to ${\rm{\Delta }}{T}_{w}^{c}$ and Δ*T* to obtain

30 $${Q}_{c}=-\frac{{\xi }_{c}{T}^{\alpha }({r}_{1}+{r}_{2}{r}_{3}{N}^{\gamma })}{{r}_{1}}{\rm{\Delta }}{T}_{w}^{c}+{\mathscr{O}}\,({\rm{\Delta }}{T}_{w}^{c}{\rm{\Delta }}T,{\rm{\Delta }}{T}_{w}^{{c}^{2}}).$$

According to Eqs (27)–(30) as well as ${\rm{\Delta }}{T}_{w}^{h}={T}_{h}-{T}_{hw}$ and ${\rm{\Delta }}{T}_{w}^{c}={T}_{cw}-{T}_{c}$, we can rewrite Eqs (29) and (30) as

31 $${Q}_{h}=\frac{{\xi }_{c}{T}^{\alpha }}{{r}_{1}}(\frac{1}{2}-\chi )\,(1+{r}_{3}{N}^{\gamma })\,({\rm{\Delta }}T-{\rm{\Delta }}{T}_{w}),$$

32 $${Q}_{c}=-\frac{{\xi }_{c}{T}^{\alpha }}{{r}_{1}}(\frac{1}{2}+\chi )\,({r}_{1}+{r}_{2}{r}_{3}{N}^{\gamma })\,({\rm{\Delta }}T-{\rm{\Delta }}{T}_{w}),$$

respectively. Inserting ${T}_{h}=T+\frac{{\rm{\Delta }}T}{2}$ and ${T}_{c}=T-\frac{{\rm{\Delta }}T}{2}$ as well as Eqs (27) and (28) into Eq. (18), and expanding it with respect to Δ*T* and Δ*T*_*w*_, Eq. (18) reduces to the simple form

33 $$\eta =\frac{1}{2}(\frac{{\rm{\Delta }}T}{T}+\frac{{\rm{\Delta }}{T}_{w}}{T})+{\mathscr{O}}\,({\rm{\Delta }}{T}^{2},{\rm{\Delta }}{T}_{w}{\rm{\Delta }}T,{\rm{\Delta }}{T}_{w}^{2}).$$

If we put together Eqs (31) and (33), we can obtain the work output *W* as

34 $$W={Q}_{h}\eta =\frac{{\xi }_{c}{T}^{\alpha }}{2{r}_{1}}(\frac{1}{2}-\chi )\,(\frac{{\rm{\Delta }}T}{T}+\frac{{\rm{\Delta }}{T}_{w}}{T})\,(1+{r}_{3}{N}^{\gamma })\,({\rm{\Delta }}T-{\rm{\Delta }}{T}_{w}).$$

It follows, using the condition of .., that the maximum work is reached at

35 $${\rm{\Delta }}{T}_{w}^{\ast }=0.$$

As expected, inserting ${\rm{\Delta }}{T}_{w}^{\ast }=0$ into Eqs (27) and (28) reproduces the optimal relation ${T}_{hw}^{\ast }={T}_{cw}^{\ast }$ [see Eq. (14)]. By instituting Eq. (35) into Eq. (33), one immediately obtains the maximum-work efficiency

36 $${\eta }^{m{\rm{w}}}=\frac{{\rm{\Delta }}T}{2T}={\eta }_{CA}+{\mathscr{O}}\,({\rm{\Delta }}{T}^{2}),$$

which is identical to a reported universal upper bound of the maximum-power efficiency from a strong-coupling Carnot-like heat engine^34^. This result derived from the reversible heat engine performing maximum work is quite general, as it is independent of the properties of the working substance and types of the engine model. It is also implied that the cyclic heat engine might satisfy the tight-coupling condition, which thus deserves our further study in the following subsection.

#### Maximum power efficiency based on linear irreversible thermodynamics

To reveal the linkage between maximum-work-reversible cycles and maximum-power-irreversible ones, we now briefly discuss the maximum-power efficiency within the context of linear irreversible thermodynamics, mapping these cyclic engines into irreversible ones. Let us consider the entropy production rate $\dot{\sigma }$ through appropriate identification of the thermodynamic fluxes and their corresponding forces. Since the entropy variation of working substance is vanishing after a whole cycle, the entropy production rate $\dot{\sigma }$ can be expressed as the sum of the entropy increase rate of two heat reservoirs,

37 $$\dot{\sigma }=-(\frac{{\dot{Q}}_{h}}{{T}_{h}}+\frac{{\dot{Q}}_{c}}{{T}_{c}})=-\frac{\dot{W}}{{T}_{c}}+{\dot{Q}}_{h}\,(\frac{1}{{T}_{c}}-\frac{1}{{T}_{h}})\simeq -\frac{\dot{W}}{T}+{\dot{Q}}_{h}\frac{{\rm{\Delta }}T}{{T}^{2}}.$$

It follows, considering Eqs (31) and (34), that Eq. (37) can be re-expressed as

38 $$\dot{\sigma }=-\frac{{\dot{Q}}_{h}}{2T}\,(\frac{{\rm{\Delta }}T}{T}+\frac{{\rm{\Delta }}{T}_{w}}{T})+{\dot{Q}}_{h}\frac{{\rm{\Delta }}T}{{T}^{2}}={J}_{s}{X}_{s}+{J}_{t}{X}_{t},$$

where the entropy and thermal fluxes are defined as

39 $${J}_{s}=\frac{{\dot{Q}}_{h}}{T},\,{J}_{t}={\dot{Q}}_{h},$$

with the affinities ${X}_{s}=-\frac{1}{2}\,(\frac{{\rm{\Delta }}T}{T}+\frac{{\rm{\Delta }}{T}_{w}}{T})$, ${X}_{t}=\frac{{\rm{\Delta }}T}{{T}^{2}}$. The two fluxes are proportional to each other, namely,

40 $${J}_{t}=T{J}_{s}.$$

These fluxes and affinities satisfy the linear constitutive relations:

41 $${J}_{s}={L}_{ss}{X}_{s}+{L}_{sq}{X}_{t},\,{J}_{t}={L}_{st}{X}_{s}+{L}_{tt}{X}_{t},$$

where the Onsager coefficients satisfy ${L}_{st}={L}_{ts},{L}_{tt},{L}_{ss}\ge 0$, and ${L}_{ss}{L}_{tt}\ge {L}_{st}{L}_{ts}$. Introducing the coupling strength parameter $q={L}_{st}/\sqrt{{L}_{tt}{L}_{ss}}$ with $|q|\le 1$ into Eq. (41), the thermal flux can be expressed in terms of the parameter *q*,

42 $${J}_{t}=\frac{{L}_{ts}}{{L}_{ss}}{J}_{s}+{L}_{tt}\,\mathrm{(1}-{q}^{2})\,{X}_{t}.$$

Combination of Eq. (40) with Eq. (42) indicates that any cyclic heat-engine model (without heat leakage) we discussed satisfies tight-coupling condition |*q*| = 1.

From Eqs (31), (34), (38) and (39), we note that the power and the efficiency are given by $\dot{W}=-{J}_{s}{X}_{s}T$ and $\eta =-\,({J}_{s}{X}_{s}T)/{J}_{t}$, respectively. It then follows, maximizing the power by setting $\partial \dot{W}/\partial {X}_{s}=0$, that the maximum-power efficiency becomes

43 $${\eta }^{mp}=\frac{{\rm{\Delta }}T}{2T}\frac{{q}^{2}}{1-{q}^{2}},$$

achieving its maximum value when and only when the tight-coupling condition (|*q*| = 1) is satisfied. Notice that, as mentioned above, the cyclic heat engine throughout the paper satisfies the tight-coupling condition |*q*| = 1, we reproduce the expression of the maximum-power efficiency ${\eta }^{mp}={\eta }^{m{\rm{w}}}$ [cf. Eq. (36)].

## Discussion

A key extension of our approach is to discuss an endoreversible heat engine in which there are multiple heat-transfer laws affected simultaneously and the irreversibility merely arises from heat fluxes between the working substance and the heat reservoirs. Without loss of generality, the heat absorbed by the system during any heat-exchange process can be given by

44 $${Q}_{\kappa }={{\mathscr{C}}}_{\kappa }^{\mathrm{(1)}}\,[{T}_{\kappa }^{\alpha }-{T}_{\kappa w}^{\alpha }]\,{\tau }_{\kappa }+{{\mathscr{C}}}_{\kappa }^{\mathrm{(2)}}\,[{T}_{\kappa }^{\beta }-{T}_{\kappa w}^{\beta }]\,{\tau }_{\kappa },$$

where *α* and *β* are two independent parameters, not restricted to our model where *β* = *α* − 1, and ${{\mathscr{C}}}_{\kappa }^{(j)}$ (*j* = 1, 2) is the heat conductivity for a given heat-transfer process *κ* with *κ* = *c*, *h*. Here *α* as well as *β* is a real number depicting a concrete heat-transfer law, and ${{\mathscr{C}}}_{\kappa }^{\mathrm{(1)}}$ (${{\mathscr{C}}}_{\kappa }^{\mathrm{(2)}}$) is the heat conductance of a heat-transfer process. For instance, in a heat-transfer process where the heat exchange is contributed by both radiation and Newton’s law conduction, we adopt *α* and *β* = 4 in Eq. (44).

For a two-heat-source cyclic heat engine, the work output *W* is given by

45 $$W={{\mathscr{C}}}_{h}^{\mathrm{(1)}}\,[{T}_{h}^{\alpha }-{T}_{hw}^{\alpha }]\,{\tau }_{h}+{{\mathscr{C}}}_{h}^{\mathrm{(2)}}\,[{T}_{h}^{\beta }-{T}_{hw}^{\beta }]\,{\tau }_{h}+{{\mathscr{C}}}_{c}^{\mathrm{(1)}}\,[{T}_{c}^{\alpha }-{T}_{cw}^{\alpha }]\,{\tau }_{c}+{{\mathscr{C}}}_{c}^{\mathrm{(2)}}\,[{T}_{c}^{\beta }-{T}_{cw}^{\beta }]\,{\tau }_{c}.$$

Since the cycle is endoreversible, we have the following constraint

46 $${\rm{\Delta }}{S}_{cycle}=0=\frac{{{\mathscr{C}}}_{h}^{\mathrm{(1)}}\,[{T}_{h}^{\alpha }-{T}_{hw}^{\alpha }]\,{\tau }_{h}+{{\mathscr{C}}}_{h}^{\mathrm{(2)}}\,[{T}_{h}^{b}-{T}_{hw}^{b}]\,{\tau }_{h}}{{T}_{hw}}+\frac{{{\mathscr{C}}}_{c}^{\mathrm{(1)}}\,[{T}_{c}^{\alpha }-{T}_{cw}^{\alpha }]\,{\tau }_{c}+{{\mathscr{C}}}_{c}^{\mathrm{(2)}}\,[{T}_{c}^{\beta }-{T}_{cw}^{\beta }]\,{\tau }_{c}}{{T}_{cw}},$$

and the efficiency $\eta =1+{Q}_{c}/{Q}_{h}$ becomes

47 $$\eta =1-\frac{{T}_{cw}}{{T}_{hw}}.$$

Substituting Eq. (46) into Eq. (13) and using the condition $\frac{\partial L}{\partial {T}_{hw}}=0$ and $\frac{\partial L}{\partial {T}_{cw}}=0$, we arrive at

48 $$\frac{{T}_{hw}\,({{\mathscr{C}}}_{h}^{\mathrm{(1)}}{T}_{hw}^{\alpha }\alpha +{{\mathscr{C}}}_{h}^{\mathrm{(2)}}{T}_{hw}^{\beta }\beta )}{{T}_{cw}\,({{\mathscr{C}}}_{c}^{\mathrm{(1)}}{T}_{hw}^{\alpha }\alpha -{{\mathscr{C}}}_{c}^{\mathrm{(2)}}{T}_{cw}^{\beta }\beta )}\frac{{{\mathscr{C}}}_{c}^{\mathrm{(1)}}\,[{T}_{c}^{\alpha }+{T}_{cw}^{\alpha }(\alpha -\mathrm{1)]}+{{\mathscr{C}}}_{c}^{\mathrm{(2)}}\,[{T}_{c}^{\beta }+{T}_{cw}^{\beta }(\beta -\mathrm{1)]}}{{{\mathscr{C}}}_{h}^{\mathrm{(1)}}\,[{T}_{h}^{\alpha }+{T}_{hw}^{\alpha }(\alpha -\mathrm{1)]}+{{\mathscr{C}}}_{h}^{\mathrm{(2)}}\,[{T}_{h}^{\beta }+{T}_{hw}^{\beta }(\beta -\mathrm{1)]}}=1,$$

which determines the optimal relation between ${T}_{hw}^{\ast }$ and ${T}_{cw}^{\ast }$. In the case when the heat transfer obeys Newton’s law, i.e., *α* = 1 as well as ${{\mathscr{C}}}_{\kappa }^{\mathrm{(2)}}=0$ (*κ* = *c*, *h*), we obtain

49 $$\frac{{T}_{cw}}{{T}_{hw}}=\sqrt{\frac{{T}_{c}}{{T}_{h}}}$$

which, together with Eq. (47), yields the maximum-work efficiency,

50 $${\eta }^{mw}={\eta }_{CA}=1-\sqrt{\frac{{T}_{c}}{{T}_{h}}}.$$

We now continue to analyze the finite-time performance of the endoreversible heat engine, applying our approach directly. Assuming that the adiabatic processes are instantaneous, we can write the total time of the cycle as $\tau ={\tau }_{c}+{\tau }_{h}=({{\rm{k}}}_{c}+{{\rm{k}}}_{h})\,\tau$, where ${{\rm{k}}}_{c}={\tau }_{c}/\tau$ and ${{\rm{k}}}_{h}={\tau }_{h}/\tau$ (with k_*c*_ + k_*h*_ = 1) define the fractional contact times with the cold and hot reservoirs, respectively.

Directly applying our approach, we insert ${T}_{h}=T+\frac{{\rm{\Delta }}T}{2}$, ${T}_{c}=T-\frac{{\rm{\Delta }}T}{2}$, ${T}_{hw}={T}_{w}+\frac{{\rm{\Delta }}{T}_{w}}{2}$ and ${T}_{cw}={T}_{w}-\frac{{\rm{\Delta }}{T}_{w}}{2}$, ${T}_{w}={\chi }_{1}^{^{\prime} }+{\chi }_{2}^{^{\prime} }{\rm{\Delta }}T+{\chi }_{3}^{^{\prime} }{\rm{\Delta }}{T}_{w}$, with ${\rm{\Delta }}{T}_{w}={T}_{hw}-{T}_{cw}$ and ${T}_{w}=\frac{{T}_{hw}+{T}_{cw}}{2}$, into Eq. (46), and then expand it with respect to Δ*T* and Δ*T*_*w*_ to obtain

51 $$\begin{array}{rcl}{\chi }_{1}^{^{\prime} } & = & T,\\ {\chi }_{2}^{^{\prime} } & = & \chi ^{\prime} =\frac{1}{2}\frac{{T}^{\alpha }\alpha \,({{\mathscr{C}}}_{h}^{\mathrm{(1)}}{{\rm{k}}}_{h}-{{\mathscr{C}}}_{c}^{\mathrm{(1)}}{{\rm{k}}}_{c})+{T}^{\beta }\beta \,({{\mathscr{C}}}_{h}^{\mathrm{(2)}}{{\rm{k}}}_{h}-{{\mathscr{C}}}_{c}^{\mathrm{(2)}}{{\rm{k}}}_{c})}{{T}^{\alpha }\alpha \,({{\mathscr{C}}}_{h}^{\mathrm{(1)}}{{\rm{k}}}_{h}+{{\mathscr{C}}}_{c}^{\mathrm{(1)}}{{\rm{k}}}_{c})+{T}^{\beta }\beta \,({{\mathscr{C}}}_{h}^{\mathrm{(2)}}{{\rm{k}}}_{h}+{{\mathscr{C}}}_{c}^{\mathrm{(2)}}{{\rm{k}}}_{c})},\\ {\chi }_{3}^{^{\prime} } & = & -\chi ^{\prime} =-\frac{1}{2}\frac{{T}^{\alpha }\alpha \,({{\mathscr{C}}}_{h}^{\mathrm{(1)}}{{\rm{k}}}_{h}-{{\mathscr{C}}}_{c}^{\mathrm{(1)}}{{\rm{k}}}_{c})+{T}^{\beta }\beta \,({{\mathscr{C}}}_{h}^{\mathrm{(2)}}{{\rm{k}}}_{h}-{{\mathscr{C}}}_{c}^{\mathrm{(2)}}{{\rm{k}}}_{c})}{{T}^{\alpha }\alpha \,({{\mathscr{C}}}_{h}^{\mathrm{(1)}}{{\rm{k}}}_{h}+{{\mathscr{C}}}_{c}^{\mathrm{(1)}}{{\rm{k}}}_{c})+{T}^{\beta }\beta \,({{\mathscr{C}}}_{h}^{\mathrm{(2)}}{{\rm{k}}}_{h}+{{\mathscr{C}}}_{c}^{\mathrm{(2)}}{{\rm{k}}}_{c})},\end{array}$$

yielding the simple form,

52 $${T}_{w}=T+\chi ^{\prime} {\rm{\Delta }}T-\chi ^{\prime} {\rm{\Delta }}{T}_{w}.$$

This, with equations ${T}_{hw}={T}_{w}+\frac{{\rm{\Delta }}{T}_{w}}{2}$ and ${T}_{cw}={T}_{w}-\frac{{\rm{\Delta }}{T}_{w}}{2}$, gives rise to

53 $${T}_{hw}=T+\chi ^{\prime} {\rm{\Delta }}T+(\frac{1}{2}-\chi ^{\prime} )\,{\rm{\Delta }}{T}_{w},$$

54 $${T}_{cw}=T+\chi ^{\prime} {\rm{\Delta }}T-(\frac{1}{2}+\chi ^{\prime} )\,{\rm{\Delta }}{T}_{w}.$$

Substituting ${T}_{hw}={T}_{h}-{\rm{\Delta }}{T}_{w}^{h}$, ${T}_{h}=T+\frac{{\rm{\Delta }}T}{2}$ and ${T}_{c}=T-\frac{{\rm{\Delta }}T}{2}$, with ${\rm{\Delta }}T={T}_{h}-{T}_{c}$ and $T=\frac{{T}_{h}+{T}_{c}}{2}$, into Eq. (44), we find *Q*_*h*_ by expanding Eq. (44) with respect to ${\rm{\Delta }}{T}_{w}^{h}$ and Δ*T* to be

55 $${\dot{Q}}_{h}={{\rm{k}}}_{h}({{\mathscr{C}}}_{h}^{\mathrm{(1)}}{T}^{\alpha -1}\alpha +{{\mathscr{C}}}_{h}^{\mathrm{(2)}}{T}^{\beta -1}\beta ){\rm{\Delta }}{T}_{w}^{h}+{\mathscr{O}}({\rm{\Delta }}{T}_{w}^{h}{\rm{\Delta }}T,{\rm{\Delta }}{T}_{w}^{{h}^{2}})\mathrm{.}$$

Similarly, directly inserting ${T}_{cw}={T}_{c}+{\rm{\Delta }}{T}_{w}^{c}$, ${T}_{h}=T+\frac{{\rm{\Delta }}T}{2}$ and ${T}_{c}=T-\frac{{\rm{\Delta }}T}{2}$ into Eq. (44), we expand Eq. (44) with respect to ${\rm{\Delta }}{T}_{w}^{c}$ and Δ*T* to obtain

56 $${\dot{Q}}_{c}=-{{\rm{k}}}_{c}\,({{\mathscr{C}}}_{c}^{\mathrm{(1)}}{T}^{\alpha -1}\alpha +{{\mathscr{C}}}_{c}^{\mathrm{(2)}}{T}^{\beta -1}\beta )\,{\rm{\Delta }}{T}_{w}^{c}+{\mathscr{O}}\,({\rm{\Delta }}{T}_{w}^{c}{\rm{\Delta }}T,{\rm{\Delta }}{T}_{w}^{{c}^{2}}).$$

According to Eqs (53)–(56) as well as ${\rm{\Delta }}{T}_{w}^{h}={T}_{h}-{T}_{hw}$ and ${\rm{\Delta }}{T}_{w}^{c}={T}_{cw}-{T}_{c}$, we can rewrite Eqs (55) and (56) as

57 $${\dot{Q}}_{h}={{\rm{k}}}_{h}\,({{\mathscr{C}}}_{h}^{\mathrm{(1)}}{T}^{\alpha -1}\alpha +{{\mathscr{C}}}_{h}^{\mathrm{(2)}}{T}^{\beta -1}\beta )\,(\frac{1}{2}-\chi ^{\prime} )\,({\rm{\Delta }}T-{\rm{\Delta }}{T}_{w}),$$

58 $${\dot{Q}}_{c}=-{{\rm{k}}}_{c}\,({{\mathscr{C}}}_{c}^{\mathrm{(1)}}{T}^{\alpha -1}\alpha +{{\mathscr{C}}}_{c}^{\mathrm{(2)}}{T}^{\beta -1}\beta )\,(\frac{1}{2}+\chi ^{\prime} )\,({\rm{\Delta }}T-{\rm{\Delta }}{T}_{w}),$$

respectively.

Inserting Eqs (53) and (54) into Eq. (47), and expanding it with respect to Δ*T* and Δ*T*_*w*_, Eq. (47) reduces to the simple form

59 $$\eta =\frac{{\rm{\Delta }}{T}_{w}}{T}+{\mathscr{O}}\,({\rm{\Delta }}{T}^{2},{\rm{\Delta }}{T}_{w}{\rm{\Delta }}T,{\rm{\Delta }}{T}_{w}^{2}).$$

If we put together Eqs (57) and (59), we can obtain the power output *P* as

60 $$P={\dot{Q}}_{h}\eta ={{\rm{k}}}_{h}\,({{\mathscr{C}}}_{h}^{\mathrm{(1)}}{T}^{\alpha -2}\alpha +{{\mathscr{C}}}_{h}^{\mathrm{(2)}}{T}^{\beta -2}\beta )\,(\frac{1}{2}-\chi ^{\prime} )\,{\rm{\Delta }}{T}_{w}\,({\rm{\Delta }}T-{\rm{\Delta }}{T}_{w}).$$

Using the condition of $\partial P/\partial {\rm{\Delta }}{T}_{w}=0$, we find that the maximum power is reached at

61 $${\rm{\Delta }}{T}_{w}^{\ast }=\frac{{\rm{\Delta }}T}{2}.$$

By instituting Eq. (61) into Eq. (59), one immediately obtains the maximum-power efficiency

62 $${\eta }^{mp}=\frac{{\rm{\Delta }}T}{2T}={\eta }_{CA}+{\mathscr{O}}\,({\rm{\Delta }}{T}^{2}).$$

This maximum-power efficiency is identical to that derived from the heat engine based on Newton’s heat transfer law^23^, but it is valid in a broader context in which the general heat transfer law in Eq. (44) is employed.

As another key extension, our approach can be directly used to describe the thermodynamics of a finite-work heat engine working with some particular systems of ultrocold fermions. One typical example is the heat engine using a free electronic Fermi gas at very low temperatures. At very low temperatures ($T\ll {T}_{F}$ with *T*_*F*_ the Fermi temperature) the heat capacity of the free electron Fermi gas can be expressed as the sum of electron and phonon contributions: $C={C}_{ele}+{C}_{phon}={\mathscr{G}}T+{\mathscr{A}}{T}^{3}$, where ${\mathscr{G}}$ is Sommerfeld constant. Based on Eqs (9) and (14), and using the exact method adopted in this paper, one can find that the efficiency at maximum work has the universality, ${\eta }_{C}/2+{\eta }_{C}^{2}/8$, and that it is universally bounded from above by the CA value.

Last but not least we should note that, since the heat capacity of the interacting system composed of atoms (e.g., the interacting Bose system^56^ or Bose-Fermi mixture system^57^) takes the form similar to Eq. (3), applying our approach will definitely yield the same conclusion about the (*α* – independent) universal behavior of the maximum-work efficiency.

## Summary

We have derived the expression of maximum-work efficiency *η* ^*m*w^ which depends on the dimensionality and form of the trapping potential, and the particle number. We showed that, at small relative temperature differences, the maximum-work efficiency can be given by, ${\eta }^{mw}={\eta }_{C}/2+({a}_{0}+1/8)\,{\eta }_{C}^{2}+\cdots$, the same as the CA efficiency *η*_*CA*_, where *a*_0_ is not strongly dependent on the system size and becomes zero in the symmetric case. This universality holds independently of the dimensionality and form of the trapping potential, the particle number, and even the strength of interaction between particles (for the weakly interacting system). Starting from the analysis of the linkage between the temperatures of the working substance and heat reservoirs, we found that, if only accurate to the first order of *η*_*C*_, the maximum-work efficiency is ${\eta }^{m{\rm{w}}}={\eta }_{CA}+O\,({\eta }_{C}^{2})$. In particular, we showed, within the context of linear irreversible thermodynamics, that the cyclic heat engine (with no heat leakage) satisfies the tight-coupling condition and the maximum-power efficiency reads ${\eta }^{mp}={\eta }_{CA}+O\,({\rm{\Delta }}{T}^{2})$. We have not considered the effects induced by the phase transition^58^ on the performance of a cyclic heat engine working in the regime of maximum work or power, which as a natural extension of this work deserves a deeper study in future.
